# Rethinking pharmaceutical development: A not-for-profit model to address global health inequities

**DOI:** 10.1016/j.fhj.2025.100255

**Published:** 2025-06-30

**Authors:** Mark Sullivan

**Affiliations:** a Medicines Development for Global Health, Melbourne, Australia; b The Kirby Institute, University of New South Wales, Sydney, Australia

**Keywords:** Pharmaceuticals, Health equity, Market failure, PDP, NTD, Not-for-profit pharmaceuticals

## Abstract

Modern pharmaceuticals represent some of the greatest achievements in science and economic development. Yet, despite significant progress, profound inequities in access to essential medicines persist, particularly in low- and middle-income countries (LMICs). Traditional market-driven pharmaceutical models have succeeded where strong commercial incentives exist, but have failed to deliver for diseases with limited market potential, particularly in the neglected tropical diseases. This article outlines the reasons for inequity within the current system, the role of not-for-profit pharmaceutical development, and the urgent need for a new, complementary model for access that centres on public health priorities rather than market return.

## Introduction

Modern, evidence-based pharmaceuticals are among the most impressive achievements of science and economic development, with many of humanity’s previously intractable and debilitating diseases now identifiable, preventable and/or treatable, resulting in longer and better lives for billions of people.^1^, ^2^, ^3^ Research and development are very different disciplines: the pharmaceutical industry fulfils an important role in research of new medicines, but it is in the breathtakingly complex, risky and expensive development process where the role of the pharmaceutical industry is essential. Medicines that address public health needs have been, broadly, successfully developed and supplied by the pharmaceutical industry for diseases where there is a strong market opportunity. However, commercial determinants of which medicines are developed and supplied has failed us in disease areas with limited market potential, leading to widespread health inequity. A not-for-profit pharmaceutical industry driven by public health need should be an obvious alternative, and yet it is nascent and under-developed.

## The economics and risks of pharmaceutical development

The barrier to such an approach to address health inequity in access to medicines is, of course, financial. The diversity of scientific expertise required to develop a new medicine, and the need to manage the substantial cost^4^ and risk inherent in the process with experience,^5^ have led to the specialisation of the pharmaceutical industry to undertake this role, almost exclusively. The likelihood of ultimately achieving regulatory approval for products in the research stages (pre-clinical) approximates to <1 %, and for those entering clinical trials, <10 %.^6^^,^^7^ DiMasi *et al*^5^ reported in 2014 that the mean cost of clinical development per approved drug was US$340 million. The capitalised cost (including the time value of money) rose to US$1.4 billion excluding the cost of failed candidates, and US$2.56 billion when these are factored in. The financial support of development requires specialist investors understanding the risks and high capital needs: in 2024, venture capital investment in pharmaceuticals was approximately US$24 billion globally,^8^ while industry invested well over US$100 billion directly.^9^ The rewards can be substantial: for example, pembrolizumab and semaglutide-based therapies achieved sales of around US$25 billion^10^ and US$21.58 billion^11^ in 2024, respectively. The 2024 pharmaceutical market is estimated to be US$1,645.75 billion.^12^

## The consequences of market failure

When market incentives are absent, the consequences for research, development and access to medicines are profound. Access to medicines is a basic human right,^13^ and yet an estimated 2 billion people in low- and middle-income countries (LMICs) lack access to essential medicines.^14^ Even in high-income countries, diseases with weaker commercial markets, such as those for antimicrobials or rare paediatric diseases, struggle under the market-driven shortcomings.

The case of the 21 neglected tropical diseases (NTDs), malaria and tuberculosis (TB) is a particularly stark example of systemic failure across the pharmaceutical value chain. Despite affecting over 1 billion people, NTDs remain largely overlooked due to insufficient investment,^15^ limited capacity for regulatory-standard development in resource-limited settings, and significant barriers to access. R&D spend in 2023 for the neglected diseases was approximately US$4 billion, with US$2.9 billion for HIV, TB and malaria,^16^ leaving approximately US$1 billion for 21 NTDs. Only 13 new pharmaceuticals targeting NTDs, malaria and TB have been approved by the US FDA since 2008,^17^ compared to over 1,000 new chemical entities for other conditions in the same period.^18^ The product pipeline is constrained by limited investment from the outset, and for those few candidates that advance beyond research, development entails the same regulatory complexity as other commercial medicines, but often lacks champions with the expertise and resources to navigate the development process. As a specific example, Chagas disease, caused by the parasite *Trypanosoma cruzi* and transmitted by triatomine bugs, affects an estimated 6 million people, mostly in Latin America, and can lead to severe cardiac and gastrointestinal complications.^19^ Treatment is with benznidazole (first registered in 1971) or nifurtimox (first registered in 1965), both oral antiparasitic drugs requiring 30–60 days of therapy. Effectiveness diminishes in chronic cases, and side effects can limit adherence. There is no vaccine, and access to diagnosis and treatment remains limited, particularly in non-endemic, resource-limited settings. A further example is scabies. It is estimated that approximately 200 million people are infected with *Sarcoptes scabiei*^20^ and yet the most recent of the topical acaricides available in LMICs is over 50 years old, and in the case of sulphur-containing treatments, well over 100 years old. Oral treatment with ivermectin is possible in some jurisdictions, but all treatments are limited by poor compliance with repeated treatments (including the need to treat all household members and contacts), and lack of ovicidal effect. These are tractable diseases to innovative treatments, and yet the lack of market opportunity has suppressed pharmaceutical development profoundly.

Even after approval, access to medicines is impeded by a lengthy and resource-intensive process that includes one or more of World Health Organization (WHO) pre-qualification, essential medicine listing, guideline inclusion, national regulatory approvals, supply chain set-up, additional clinical trials and community-level engagement, which can add years to timelines and substantially increase costs. These downstream access barriers disincentivise upstream investment, particularly when there are expectations of donation or minimal financial return. Further, the data required by medical professionals and affected communities to determine a medicine’s relevance in their settings often differ from those required for regulatory approval. Yet, the model for producing these data is largely driven by academic groups and tends to be fragmented, underfunded and reliant on small, heterogeneous studies or meta-analyses of variable quality, posing a real risk to sound clinical decision-making. Moreover, the lack of clear product ‘ownership’, particularly for older, off-patent medicines with multiple generic manufacturers, further complicates efforts to coordinate evidence generation, monitor safety, or pursue essential improvements, such as paediatric formulations or usage guidance for pregnancy and breastfeeding.

## The role of philanthropy and investment incentives

The dependence on the financial and technical philanthropy of the pharmaceutical industry and a limited number of important financial donors in the development of new medicines is illustrative of the failure of our model to sustainably support delivery of new products. In addition to industry, key players are the Bill and Melinda Gates Foundation, the Wellcome Trust, the Japanese Global Health Innovative Technologies Fund, the Korean Right Fund, the European and Developing Countries Clinical Trials Partnership and funding governments including the Netherlands, the UK, Germany, Switzerland and Australia. Social impact investment, such as the Global Health Investment Corporation, Adjuvant Capital and AXA IM, have brought fresh funding and perspectives to the field. However, while helping to attract investment, the key incentive of the US Priority Review Voucher (PRV), established in 2008, has limitations.^21^ Further, the value of the PRV is approximately US$100 million,^22^ which, when risk adjusted, is insufficient to support development earlier than mid- to late phase 2. Without the PRV, most of the NTDs have no prospect of market return and thus have limited ability to attract social impact investment.

## The importance and challenges of product development partnerships

A further consequence of limited funding is that the skills required to execute the work are also in short supply. Product development partnerships (PDPs)^23^ are public–private, non-profit partnerships established to address these needs, working in concert with the pharmaceutical industry’s philanthropic efforts in driving the development of new pharmaceuticals and diagnostics for poverty-related and neglected diseases. They have delivered the first ever drug approved for treatment of highly drug-resistant forms of TB, a single-dose treatment to prevent relapse of *P vivax* malaria, the first new treatment for onchocerciasis in 30 years, and an oral cure for all stages of sleeping sickness. However, funding for their activities has been stagnant or falling for years,^16^ creating an existential challenge. Only two agents (moxidectin for the treatment of river blindness by Medicines Development for Global Health, and pretomanid for the treatment of TB by TB Alliance) were developed outside the typical PDP/pharmaceutical company partner model, with the pretomanid licence now held by the generics company Mylan Pharmaceuticals.

## The role of the Essential Medicines List

To highlight the importance of certain medicines for disease management, regardless of market, the WHO publishes the Essential Medicines List^24^ to identify medicines that need to be available in functioning health systems ‘at all times in adequate amounts’. However, the essential medicine system is passive, does not include a mechanism of financial support for the manufacturers to ensure continued supply of products, and does not address fair pricing and affordability directly. The list does not address major gaps that affect public health and need to be filled by innovation and R&D. Instead, it relies on the cooperation of the pharmaceutical and generics industries to produce the required medicines without guarantees of affordability and accessibility.

## A not-for-profit model for public health impact

A new model is needed to complement the existing model of developing and delivering medicines for diseases where there is little or no market incentive – one that is driven primarily by public health needs and the voices of affected communities. The approaches employed to date for development and access have contributed to significant gaps in addressing global health needs.

Not-for-profit organisations could increasingly fulfil the role of licence holder(s) responsible and accountable for the product’s research and development, manufacture, supply, and meeting regulatory obligations. The attraction is that this public health-driven approach would provide a home for medicines at the end of the R&D journey and elevate medical need in the decision-making process for both development of medicines and continued access. Examples of this are currently few: Medicines Development for Global Health is a not-for-profit pharmaceutical company focused on developing medicines for diseases that disproportionately affect LMICs and marginalised communities in high-income countries, where market incentives are weak or absent. This model, and those of other PDPs, is based on broad collaboration: pharmaceutical development, regardless of where the intended beneficiaries live, remains risky, skill dependent and capital intensive. Bringing the necessary skills to bear on important public health interventions with diverse funding sources to support the entire product lifecycle will deliver significant outcomes for billions of people. If funding comes from public sources rather than investment, the cost of R&D (and R&D failures) should not be included in the medicine’s pricing structure, increasing the ability of LMICs to afford a novel medicine. The proof of concept that a not-for-profit can undertake all of the necessary steps that a pharmaceutical industry partner would was achieved with moxidectin: in January 2025, Medicines Development for Global Health became the first not-for-profit to distribute a novel medicine without a pharmaceutical or generic partner, providing treatment in Ghana.

Given that a significant cost element both in development and access to medicines is in the cost of manufacturing, combining a not-for-profit manufacturing capability with a not-for-profit sponsor would address the major issues of development and supply of medicines where there is market failure. Curating the introduction and maintenance of any pharmaceutical (and potentially diagnostics), including pharmacovigilance, regulatory roll-out, education and medical information, supply chain management and supply forecasting is a further benefit. Charging for the medicines on a cost recovery basis is a sustainable and expandable model.

## Conclusion

Market-driven pharmaceutical development has delivered extraordinary progress but has also left critical gaps. Diseases with limited market potential remain neglected, despite affecting billions. A complementary model based on public need, collaborative expertise and diverse funding is urgently required. Not-for-profit pharmaceutical development demonstrates a viable path forward, with the potential of addressing global health inequities with sustainability and scale. The addition of not-for-profit manufacturing capability would provide the most cost-effective means of developing and supplying new products for affected communities.

## CRediT authorship contribution statement

**Mark Sullivan:** Writing – review & editing, Writing – original draft, Investigation, Conceptualization.

## Declaration of competing interest

The authors declare the following financial interests / personal relationships which may be considered as potential competing interests:

Mark Sullivan reports financial support was provided by Medicines Development for Global Health Limited. The author’s partner works for Gilead Sciences Inc. If there are other authors, they declare that they have no known competing financial interests or personal relationships that could have appeared to influence the work reported in this paper.
